# Discovery of integrons in Archaea: Platforms for cross-domain gene transfer

**DOI:** 10.1126/sciadv.abq6376

**Published:** 2022-11-16

**Authors:** Timothy M. Ghaly, Sasha G. Tetu, Anahit Penesyan, Qin Qi, Vaheesan Rajabal, Michael R. Gillings

**Affiliations:** ^1^ School of Natural Sciences, Macquarie University, Sydney, New South Wales 2109, Australia.; ^2^ ARC Centre of Excellence in Synthetic Biology, Macquarie University, Sydney, New South Wales 2109, Australia.

## Abstract

Horizontal gene transfer between different domains of life is increasingly being recognized as an important evolutionary driver, with the potential to increase the pace of biochemical innovation and environmental adaptation. However, the mechanisms underlying the recruitment of exogenous genes from foreign domains are mostly unknown. Integrons are a family of genetic elements that facilitate this process within Bacteria. However, they have not been reported outside Bacteria, and thus their potential role in cross-domain gene transfer has not been investigated. Here, we discover that integrons are also present in 75 archaeal metagenome-assembled genomes from nine phyla, and are particularly enriched among Asgard archaea. Furthermore, we provide experimental evidence that integrons can facilitate the recruitment of archaeal genes by bacteria. Our findings establish a previously unknown mechanism of cross-domain gene transfer whereby bacteria can incorporate archaeal genes from their surrounding environment via integron activity. These findings have important implications for prokaryotic ecology and evolution.

## Teaser

Integrons can facilitate horizontal gene transfer between the two domains of prokaryotes, Archaea and Bacteria.

## INTRODUCTION

Horizontal gene transfer between different domains of life can be a major driver in species evolution (*1*). There are now numerous examples of genes that have been transferred among Archaea, Bacteria, and Eukarya (*2*–*7*). Among the consequences of these gene transfers are the gain of novel biochemical functions and the ability to colonize specific environmental niches (*3*, *8*, *9*). However, the molecular mechanisms for most of these transfer events are unknown.

Integrons are genetic elements known to facilitate horizontal gene transfer within Bacteria (*10*–*13*). Integrons can capture exogenous genes, known as gene cassettes, by site-specific recombination. Gene cassette capture is mediated by an integron integrase (IntI), which catalyzes the recombination between the recombination site of the inserting cassette (*attC*) and the endogenous integron attachment site (*attI*), immediately adjacent to the *intI* gene. Multiple gene cassettes can be inserted within a single integron, forming cassette arrays that range from 1 to more than 200 sequential cassettes (*10*, *12*). Integrons are mostly known for their role in driving the global antibiotic resistance crisis by disseminating diverse resistance determinants among bacterial pathogens (*14*–*16*). However, it is now clear that integrons play a much broader role in bacterial evolution and niche adaptation (*17*). The functions encoded by integron gene cassettes are extraordinarily diverse and extend far beyond those of clinical relevance (*13*, *18*, *19*).

To date, integrons have only been found within bacterial genomes, where they have been detected within diverse phyla (*20*). However, gene cassette amplicon sequencing has yielded cassette-encoded proteins that share homology with archaeal proteins (*21*, *22*). Without broader genomic context, however, the taxonomic residence of these gene cassettes is unknown.

Here, we screened all publicly available archaeal genomes to show that integrons are not only limited to Bacteria but also present in Archaea. Archaeal integrons exhibit the same characteristics and functional components as bacterial integrons. Furthermore, we demonstrate experimentally that diverse archaeal gene cassettes can be successfully recruited by a bacterial host, facilitated by integron-mediated recombination. Such a mechanism can thus permit bacteria to recruit archaeal gene cassettes present in their surrounding environment, with important implications for prokaryotic evolution.

## RESULTS AND DISCUSSION

### Discovery of integrons in Archaea

**Fig. 1.**
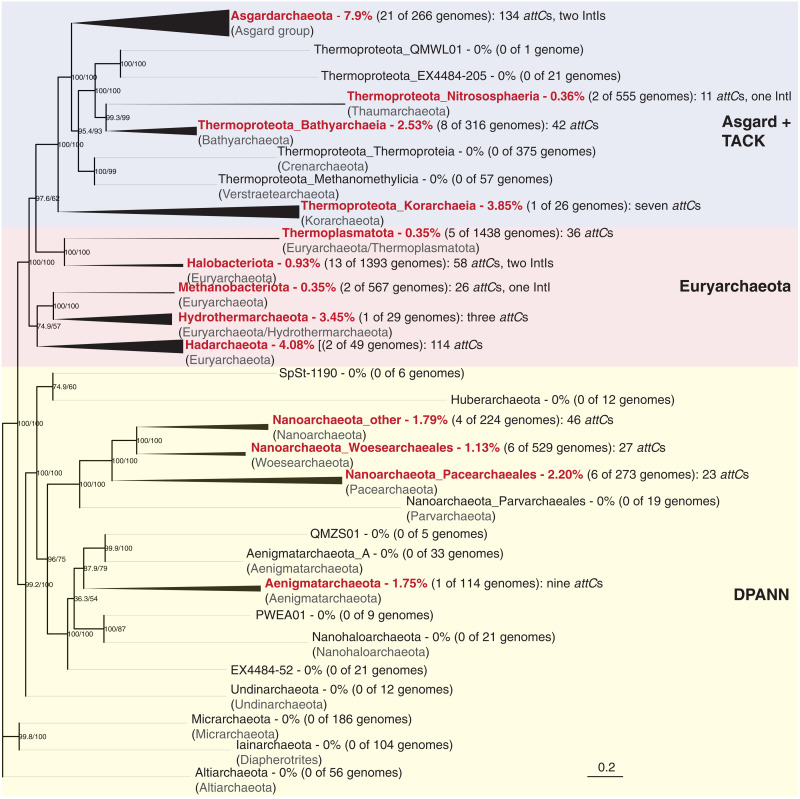
Phylogenetic distribution of integrons among Archaea. Archaeal taxa found to carry integrons are labeled in red. Branch thickness indicates the proportion of genomes with integrons for each taxon. The phylogeny is based on a concatenated alignment of 53 top-ranked marker proteins recently identified to better recover monophyletic archaeal lineages (*55*). A maximum likelihood phylogenetic tree was inferred with the LG+C60+F+R model with an ultrafast bootstrap approximation (left node label) and Shimodaira–Hasegawa-like approximate likelihood ratio test (right node label), each run with 1000 replicates. The tree was artificially rooted with Altiarchaeota to agree with recent rooted archaeal phylogenies (*55*). Tips are labeled using the Genome Taxonomy Database (GTDB; top) and National Center for Biotechnology Information (NCBI; bottom) taxonomic nomenclature. To show major NCBI phyla collapsed under GTDB taxonomy, the GTDB phyla Thermoproteota (TACK group) and Nanoarchaeota (DPANN group) were split into class-level and order-level groupings, respectively. The scale bar indicates the average number of substitutions per site.

Here, we report the discovery of integrons in the domain Archaea. We screened 6718 archaeal genomes for integrons using the standard criteria applied to integron surveys in Bacteria (*20*, *23*, *24*). These include the presence of IntI genes and/or clusters of gene cassette *attC*s (defined as at least two *attC*s with less than 4 kb between each). We identified integrons in 75 archaeal metagenome-assembled genomes (MAGs) from nine phyla (Fig. 1 and data S1). It is not unexpected that integrons were detected only in MAGs, given that they constituted ~95% of all available archaeal genomes. However, to ensure that these integrons did not arise from contaminating bacterial contigs, incorrectly binned with archaeal MAGs, we applied stringent MAG refinement and quality filtering (see Materials and Methods for details). In addition, we found that ~7% of integron-bearing MAGs had at least one archaeal phylogenetic marker gene on the same contig as an integron (data S2), confirming these to be located on archaeal chromosomes. No integron was ever colocated with a bacterial marker gene. The markers used for this analysis consisted of a comprehensive set of 233 marker proteins identified as suitable for phylogenetic inference (*25*).

Among the 75 archaeal genomes, we detected six IntIs and 539 *attC* sites (excluding all singleton *attC*s). We found that archaeal integrons have a patchy distribution with varying prevalence across the phylogeny of Archaea (Fig. 1). In particular, integrons were significantly enriched in the phylum Asgardarchaeota (χ^2^ test, *P* < 0.00001) (Fig. 1), being detected in almost 8% of available Asgard genomes. Asgardarchaeota contributed the most genomes with detectable integrons (28%) and the greatest number of gene cassettes (24.9%), despite having relatively few genomes among the dataset (comprising 4% of available archaeal genomes). We also detected integrons in 3 to 4% of genomes from Hadarchaeota, Hydrothermoarchaeota, and Korarchaeia (Fig. 1), although these comprised few available genomes (*n* < 50). A patchy phylogenetic distribution of integrons has similarly been observed among Bacteria (*20*). For example, in the phylum Proteobacteria, integrons are enriched within the class Gammaproteobacteria (20% of genomes) while being entirely absent from its sister class Alphaproteobacteria. This is intriguing given that integrons have been detected at widely varying prevalence in more distantly related bacterial phyla such as Cyanobacteria, Spirochaetota, Planctomycetota, Chloroflexota, Bacteroidota, and Desulfobacterota (*20*, *23*).

### Genetic structure of archaeal integrons

We found that archaeal integrons exhibit the same structure and functional components as bacterial integron cassette arrays (fig. S1). That is, tandem arrays of short open reading frames, generally in the same orientation, interspersed by *attC* recombination sites. Archaeal *attC*s exhibit the same single-stranded folding structure as bacterial *attC*s, which is essential for them to act as structure-specific DNA recombination sites (*26*–*32*). We also note that archaeal IntIs exhibit the defining characteristics of bacterial IntIs, being tyrosine recombinases that have a unique IntI-specific additional domain surrounding the patch III motif region necessary for integron-mediated recombination (*33*). We found examples of “complete” integrons, these being cassette arrays adjacent to a detectable *intI* gene (fig. S1). We also found examples of putative *attI* sites, which act as insertion points for incoming gene cassettes. These *attI*s were immediately downstream of the *intI* gene, semi-conserved across distinct archaeal phyla (fig. S2, A and B), and exhibited the same canonical insertion point as all known bacterial *attI*s (fig. S2C).

Most archaeal integrons that we identified were CALINs (clusters of *attC*s lacking IntIs; data S3). This is not unexpected given the fragmented nature of MAGs and the high prevalence of CALINs also found in bacterial genomes. Among Bacteria, CALINs are more abundant than integrons that have an *intI* gene and exhibit a much wider taxonomic distribution (*20*). Note that cassettes within CALINs can still be excised and/or captured by exogenous IntIs, either in trans or following the uptake of DNA from a lysed cell. Thus, even without IntI genes, CALINs are still ecologically and evolutionarily important. Two so-called 'In0' elements were also detected among Archaea. These are integrons that have an *intI* gene without an adjacent *attC* site (fig. S1). However, both archaeal genomes with an In0 also had clusters of *attC* sites on other contigs. Among our dataset, the longest array of *attC*s on the same contig was 12; however, we found as many as 107 *attC*s (more than 18 contigs) within a single MAG (data S1). The number of *attC*s within a single MAG ranged from 2 to 107, with an average of seven *attC*s.

### Platforms for cross-domain gene transfer

**Fig. 2.**
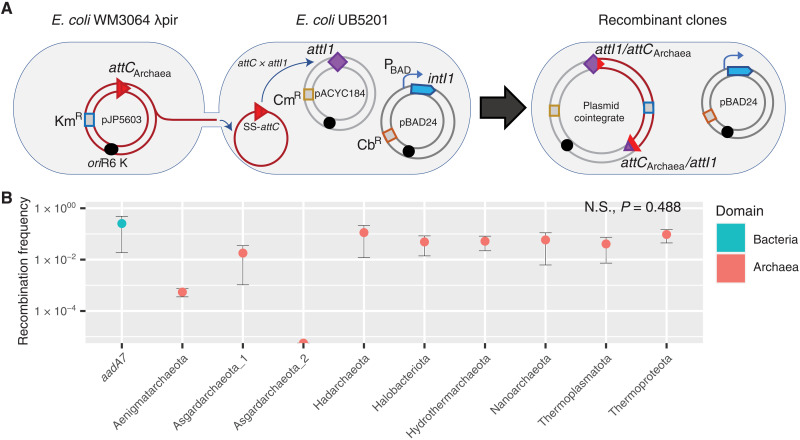
Cassette recruitment (*attC* × *attI* recombination) assays. (**A**) Schematic outlining the experimental setup of the cassette insertion assays. The kanamycin resistance (Km^R^) suicide vector pJP5603 with an *attC* site is delivered into the recipient *E. coli* UB5201 strain via conjugation. The recipient strain carries an *intI1* gene, expressed from the inducible P_BAD_ promoter, and an *attI1* site, residing on the carbenicillin resistance (Cb^R^) pBAD24 and chloramphenicol resistance (Cm^R^) pACYC184 backbones, respectively. The donor suicide vector cannot replicate within the recipient host and thus can only persist following *attC* × *attI* recombination to form a plasmid cointegrate. (**B**) Average recombination frequencies (log_10_ scale, ±1 SE) between *attI1* and nine archaeal *attC*s (with phyla of origin labeled along the *x* axis) and the paradigmatic bacterial *attC* site (*attC_aadA7_*), used as positive control. Average frequencies were calculated following three independent cassette insertion assays (see Materials and Methods for details). No statistically significant difference in recombination frequencies were detected among the tested *attC*s (Kruskal-Wallis test, *n* = 27; df = 8, *P* = 0.488). Recombination frequencies are shown for *attC* bottom strands only. See table S1 for *attC* top strand recombination frequencies. N.S., not significant.

Archaeal gene cassettes with *attC*s from diverse phyla can be recognized and recruited by Bacteria (Fig. 2). We demonstrate that cassette insertion (*attC* × *attI* recombination) can occur following the conjugation of circular DNA molecules with archaeal *attC*s into an *Escherichia coli* recipient harboring a bacterial class 1 integron (Fig. 2A). Insertion events were confirmed with Sanger sequencing of the polymerase chain reaction (PCR)–amplified *attC*/*attI* recombination junctions (Fig. 2A and fig. S3). We found that recruitment of cassettes with archaeal *attC*s occurred at similar frequencies to that of the paradigmatic bacterial *attC* site, *attC_aadA7_*, which we used as a positive control (Fig. 2B and table S1). We observed an average recombination frequency of 2.5 × 10^−1^ between *attI1* and *attC_aadA7_*. Comparable frequencies (ranging from 1.9 × 10^−4^ to 3.2 × 10^−1^, with an average of 5.1 × 10^−2^) were observed for eight of the nine archaeal *attC*s (Kruskal-Wallis test, *P* = 0.488), which were selected from multiple archaeal phyla. Furthermore, we confirmed that cassette recruitment was mediated by IntI1 activity, because no *attC* × *attI* recombination events were detected when *intI1* was absent or when its expression was suppressed (table S1). This is of significant ecological and evolutionary importance, as the uptake of gene cassettes released into the environment from a lysed archaeal cell can be readily incorporated into bacterial genomes via integron-mediated recombination. We therefore show that integrons can facilitate gene transfer between the two domains of prokaryotes.

We find that the most clinically significant class of integrons (class 1) can recruit archaeal cassettes as efficiently as bacterial cassettes. Class 1 integrons are highly promiscuous because of their association with diverse mobile genetic elements, facilitating their spread into at least 104 bacterial species from 44 genera (*13*). They collectively carry more than 130 different resistance genes (*14*), most of which are of unknown taxonomic origin (*23*). Our findings open the possibility that Archaea could be an unexplored source of class 1 integron gene cassettes. Regardless, our findings indicate that any bacterial strain with a class 1 integron has the capacity to incorporate exogenous genes from diverse archaeal phyla, greatly expanding the genetic pool that they have access to.

The cross-domain transfer of integron gene cassettes is possibly widespread. For example, we detected 23 *attC*s from six archaeal genomes that exhibited 95 to 100% nucleotide identity to *attC*s within sequenced bacterial integrons (data S4). The archaeal *attC*s were from three phyla: Nanoarchaeota, Thermoproteota, and Hadarchaeota. The homologous *attC*s in Bacteria were found in 26 genomes from five phyla: Proteobacteria, Spirochaetota, Myxococcota, Nitrospirota, and Desulfobacterota. One of these *attC* sites was associated with a class 1 integron gene cassette, encoding a reduced form of nicotinamide adenine dinucleotide phosphate–dependent oxidoreductase found on five different Enterobacteriaceae plasmids (data S4). In Archaea, however, this *attC* site was part of a cassette that encoded a ligand-binding protein of unknown function. Nevertheless, because strong *attC* homology is a characteristic of cassettes that share the same taxonomic origin (*23*, *34*, *35*), it is possible that some clinically relevant gene cassettes now found on class 1 integrons might be of archaeal origin. We also find that an archaeal cassette (Methanobacteriota; accession: GCA_020055905.1), encoding a putative addiction module, shares a 92% nucleotide homology with a bacterial cassette (Planctomycetota; accession: AP021856), suggesting that a recent common ancestral gene has transferred between the two domains.

An analysis of cassette ribosome-binding sites (RBSs) suggests that the gene cassettes observed in archaeal genomes can be expressed by taxa from both prokaryotic domains. All RBS motifs associated with archaeal integrons are present among complete archaeal and bacterial genomes, respectively (data S5). This suggests that, following cross-domain transfer, it is possible for gene cassettes to be expressed within their new archaeal or bacterial host.

### Diversity of IntIs

**Fig. 3.**
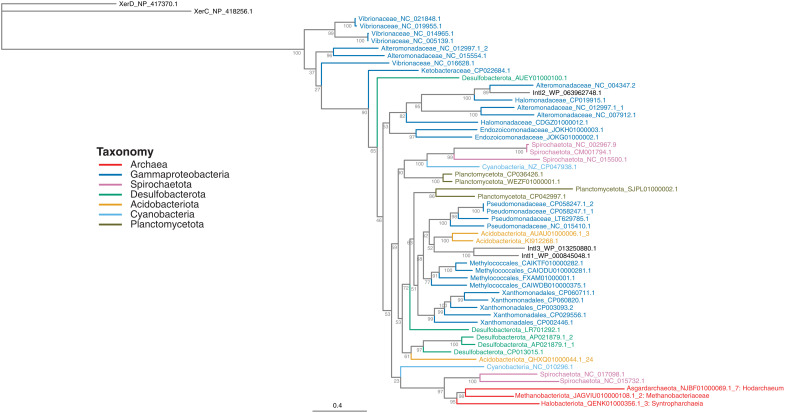
Phylogeny of IntIs from Archaea and Bacteria. To root the tree, the tyrosine recombinases XerC and XerD from *E. coli* were used as out-groups. Integron integrases (IntIs) are colored according to their taxonomy. Archaeal IntIs are also labeled with their lowest taxonomic classification. The scale bar indicates the average number of substitutions per site.

Archaeal IntIs are phylogenetically distinct from bacterial IntIs (Fig. 3). We detected six IntIs from four archaeal phyla (Fig. 1); however, three of these were excluded from further phylogenetic analysis based on either short sequence length (<200 amino acids) or partial coverage of the IntI-specific domain (fig. S4). We found that archaeal IntIs form their own monophyletic clade separate from known bacterial IntIs (*23*). This strongly suggests that IntI radiation has occurred within Archaea after a single ancient acquisition event from Bacteria. Regardless, we show that IntIs from distinct archaeal phyla, isolated from different environments, are more closely related to each other than they are to any bacterial IntI.

The closest sister clade to the archaeal IntIs comprises two Spirochaetota IntIs (Fig. 3). These two IntIs are phylogenetically distinct from “typical” Spirochaetota IntIs, which are generally in reverse orientation (*11*, *36*). Furthermore, the two Spirochaetota that harbored atypical IntIs were isolated from extreme environments: a brine layer within an alkaline lake and a hot spring, respectively; environments known to have a relatively high abundance of Archaea (*37*). Thus, these atypical Spirochaetota IntIs might have been horizontally acquired from Archaea that share the same extreme environments, although the direction of gene transfer cannot be determined with certainty.

### Diversity of *attC* recombination sites

**Fig. 4.**
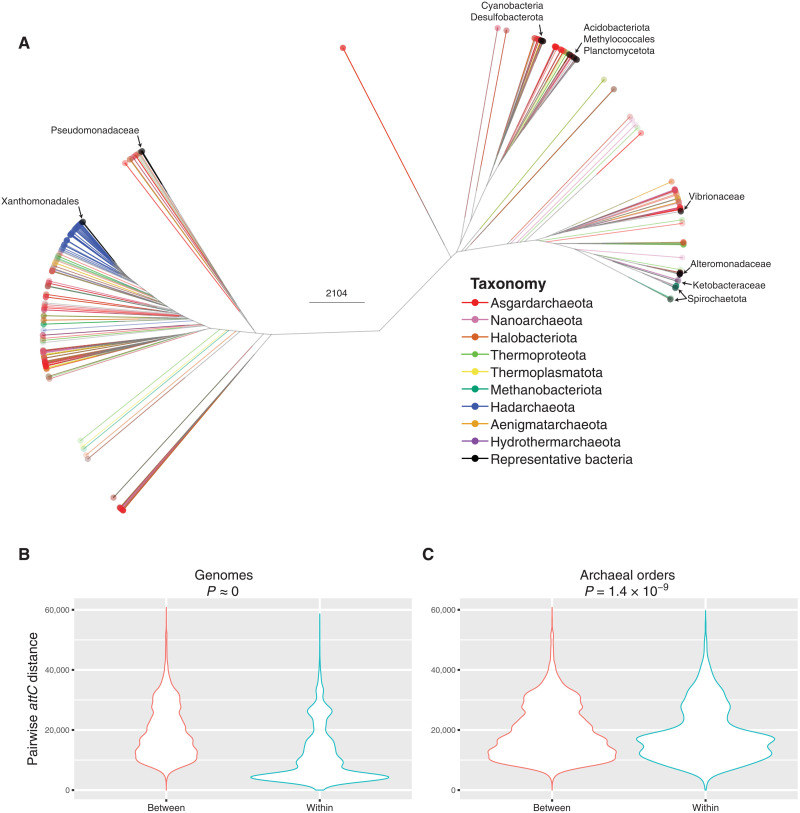
Structural and sequence diversity of archaeal *attC* recombination sites. (**A**) Structure-based clustering of all archaeal and representative bacterial *attC*s. Branches and tips are colored according to archaeal phylum. The taxa of bacterial *attC*s are labeled with arrows. The scale bar indicates pairwise *attC* alignment distances as determined by RNAclust (*61*) and LocARNA (*62*, *63*). (**B**) Distribution of the sequence and structural distances calculated for all pairwise comparisons of *attC*s within and between genomes. (**C**) Distribution of distances for all pairwise comparisons of *attC*s from different genomes that are either from the same or different archaeal orders.

Archaeal *attC*s exhibit broad sequence and structural diversity (Fig. 4A). We find that some archaeal phyla have *attC*s with a restricted diversity (e.g., Hadarchaeota and Aenigmatarchaeota), while other phyla have extremely variable *attC*s distributed throughout the *attC* diversity space (e.g., Asgardarchaeota, Nanoarchaeota, and Thermoproteota). This distribution could indicate that different taxa have different propensities for horizontal exchange of gene cassettes (*13*, *23*). We show that archaeal *attC*s are significantly more similar within a genome than between genomes (Fig. 4B). This characteristic is also a hallmark of chromosomal bacterial integrons (*20*, *35*). We also show that *attC*s are more similar between different genomes from the same archaeal order than they are between genomes from different orders (Fig. 4C). This order-level *attC* homology is also seen within Bacteria (*23*, *34*). Thus, the ecological and evolutionary forces that promote and/or constrain *attC* diversity (*13*) are likely to be similar for both Archaea and Bacteria.

There is an extensive overlap in the sequence and structural diversity of *attC*s from Archaea and Bacteria (Fig. 4A), which are regularly placed in the same clade. This provides evidence that there is a mechanistic overlap between archaeal and bacterial *attC*s and suggests that gene cassette transfer between the two domains does occur. It also suggests that the recruitment of extra domain gene cassettes can be facilitated by diverse classes of integrons, of which there are thousands [based on IntI amino acid homology (*38*)]. The broad distribution of integrons among the two domains suggests that integron-mediated transfer plays an important role in prokaryotic evolution.

### Functional diversity of gene cassettes

We detected 549 cassette-encoded proteins among Archaea. Only 23.1% of these could be classified into a known Clusters of Orthologous Groups of proteins (COG) category (fig. S5). In contrast, 47.4% of all proteins from the 75 integron-bearing archaeal genomes could be assigned a known COG category. This underrepresentation (χ^2^ test, *P* < 0.00001) of known COGs among cassette proteins has previously been reported for bacterial integrons (*10*, *11*, *34*). To gain further insight into possible cassette functions, eggNOG 5.0 (*39*) and Pfam (*40*) database searches were performed, assigning putative functions to 228 (41.5%) of the archaeal cassette–encoded proteins. Of those with functional predictions, proteins involved in toxin-antitoxin (TA) systems (10.5%), phage resistance proteins via DNA methylation or restriction endonuclease activities (8.3%), and acetyltransferases (4.4%) were particularly prevalent (data S6). These are the functions most commonly reported for gene cassettes in Bacteria (*11*, *13*, *34*, *35*, *41*). TA gene cassettes are particularly common in bacterial integrons, where they can stabilize very large cassette arrays (*42*, *43*). The antitoxin modules of TA cassettes can also counteract the toxins of homologous systems found on plasmids and phage, thus potentially protecting their host from invading mobile elements (*44*, *45*).

In addition, 13.2% of archaeal cassette–encoded proteins had signal peptides, which represents a significant enrichment relative to their broader genomic contexts (6.9%; χ^2^ test, *P* < 0.00001). Signal peptides are short amino acid tag sequences that target proteins into, or across, membranes. Again, transmembrane and secreted proteins are commonly encoded by gene cassettes in Bacteria (*34*) and are hypothesized to help facilitate interactions with their broader environment (*13*).

**Fig. 5.**
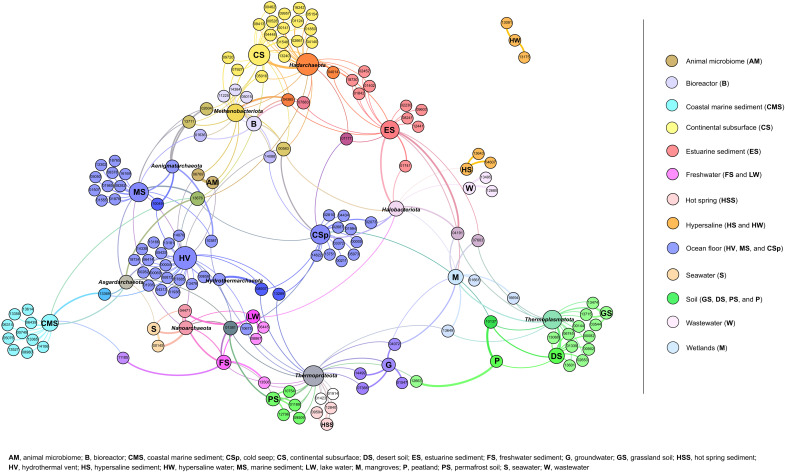
A network linking Pfam functions of archaeal integron gene cassettes with their taxonomic and environmental contexts. The force-directed representation of the network is constructed on the basis of co-occurrence patterns and correlations (*P* < 0.05) among Pfam functions, taxonomic groups, and specific environments from which the organisms were sampled. Nodes that represent taxonomic groups and specific environments are labeled accordingly. All other nodes denote Pfam functions and are labeled with a Pfam number preceded by “PF.” Specific environments are grouped into broader environment types, each of which is colored as per the panel. Pfams directly linked to specific environment types are colored in corresponding colors. Pfams linked to more than one environment type are colored in overlapping colors. The size of the node is relative to the node authority based on the degree of correlations. Edges (the lines connecting the nodes) represent correlations between nodes. Edge color denotes the overlapping color of the two nodes it connects. Edge thickness represents the strength of correlation. The full description of all correlations and Pfam functions is presented in data S7.

We find that functions of archaeal cassettes are associated with their environment (Fig. 5). Functional families cluster according to their specific environment, and these environmental clusters, in turn, group according to their broader environmental type (Fig. 5). This environmentally explicit clustering might be the result of local ecological and evolutionary forces. That is, gene cassettes in Archaea confer niche-specific functional traits and/or horizontal transfer of cassettes that occurs between archaeal phyla colocated in the same environment.

Here, we present the first evidence of integrons in the domain Archaea. We demonstrate that they have the same functional characteristics as bacterial integrons. We also present experimental evidence that bacteria can successfully recruit archaeal gene cassettes, facilitated by integron-mediated DNA recombination. Our results thus establish a novel mechanism for cross-domain gene transfer between Archaea and Bacteria, whereby integron-carrying bacteria can incorporate archaeal gene cassettes present in their surrounding environment. We also find that, although archaeal IntIs are phylogenetically distinct from bacterial IntIs, their associated *attC* recombination sites are shared with Bacteria. This suggests that integron-mediated cross-domain gene transfer might be common and could play an important role in prokaryotic evolution.

## MATERIALS AND METHODS

### Data acquisition and quality filtering

All available archaeal genomes were downloaded from the National Center for Biotechnology Information (NCBI) Assembly Database (*n* = 8160; last accessed 5 October 2021). Of these, ~95% were MAGs. We applied stringent filtering criteria to remove low-quality MAGs. First, to improve MAG quality, we identified and removed contaminating contigs from each MAG using MAGpurify v2.1.2 (*46*) with the following modules: “phylo-markers,” which finds taxonomically discordant contigs using 100 archaeal and 88 bacterial single-copy taxonomic marker genes from the PhyEco database (*47*); “clade-markers,” which finds contaminating contigs using a database of 855,764 clade-specific prokaryotic marker genes [MetaPhlAn2 database (*48*)]; “tetra-freq,” which uses principal components analysis (PCA) to identify contaminating contigs with outlier tetranucleotide frequency; and “gc-content,” which uses PCA to identify contaminating contigs with outlier GC content.

After refinement, the quality of the genomes was assessed using CheckM v1.1.3 (*49*), which uses single-copy lineage-specific marker genes to estimate genome completeness and contamination. There is strong community consensus that high-quality MAGs are those that are more than 90% complete and have less than 5% contamination, while medium-quality MAGs have a completeness of ≥50% and contamination of <10% (*25*, *46*, *50*–*53*). In this context, however, we were more concerned with the level of contamination than completeness and thus removed all genomes with an estimated contamination of ≥5%. The completeness of the remaining genomes ranged from 15 to 100%, with a median of 81%. The estimated contamination ranged from 0 to 4.98%, with a median of 0.93%.

Archaeal genomes were assigned taxonomic classifications based on the Genome Taxonomy Database (GTDB) (*50*–*52*) using GTDB-Tk v1.6.0 (*54*) with release 06-RS202 of the GTDB. We used the classify_wf command with default settings. This workflow identifies and aligns 120 bacterial and 122 archaeal phylogenetic marker genes (*25*). GTDB-Tk then classifies each genome based on its placement into domain-specific reference trees (built from 47,899 prokaryote genomes), its relative evolutionary divergence, and average nucleotide identity to reference genomes in the GTDB. Any genomes not classified within the domain Archaea were removed. This resulted in a final set of 6718 archaeal genomes retained for further analysis.

To infer the phylum-level phylogeny of Archaea, the highest quality representative genome from each phylum was selected on the basis of its genome quality score [defined by Parks *et al.* (*25*) as the estimated completeness of a genome minus five times its estimated contamination]. We inferred the phylogeny of the representative genomes using a set of 53 top-ranked marker proteins recently identified to better recover monophyletic lineages in Archaea (*55*). We used the updated version of GTDB-Tk v2.0.0 (*54*) to generate a concatenated and trimmed multiple sequence alignment of the 53 marker proteins. A maximum likelihood phylogenetic tree was generated from the alignment using IQ-TREE v1.6.12 (*56*) with the LG+C60+F+R model (parameters: -m LG+C60+F+R -bb 1000 -alrt 1000).

### Integron detection

Because of faster processing speeds of large datasets, we initially screened all filtered genomes for *attC* recombination sites using *attC*-screening.sh (*38*) (https://github.com/timghaly/integron-filtering) with default parameters. This script uses the HattCI (*57*) + Infernal (*58*) pipeline [first described by Pereira *et al*. (*24*)] to search for the conserved sequence and structure of *attC* sites. Genomes that had at least one detectable *attC* site were additionally screened using IntegronFinder v2.0rc6 (parameters: --local-max --cpu 24 --gbk) (*20*), which searches for IntIs and gene cassette arrays. Only IntIs, *attC*s, and cassette-encoded proteins identified by IntegronFinder were included in downstream analyses.

To ensure that these integrons were not from contaminating bacterial contigs that had been incorrectly binned with archaeal MAGs, we screened all contigs containing an integron for prokaryotic marker genes using GTDB-Tk v1.6.0 (*54*). These consisted of a comprehensive set of 233 proteins identified as suitable phylogenetic markers (*25*). We found a total of nine prokaryotic marker genes among seven integron-bearing contigs from five genomes. To identify the taxonomy of the marker genes, we searched for their homologs in the NCBI nr database via a BLASTP search and found that they best aligned with genes from a diverse set of archaeal genomes (excluding the specific genomes in which they derived from). None of these prokaryotic marker genes were ever identified as bacterial. See data S2 for a list of marker proteins and BLASTP hits.

### Analysis of IntIs, *attC* sites, and cassette-encoded proteins

IntegronFinder identifies IntIs using the overlap of two protein hidden Markov model profiles. The first is the Pfam profile PF00589 to identify tyrosine recombinases, and the second is a protein profile built from the IntI-specific domain that separates IntIs from other tyrosine recombinases (*33*). Identified archaeal IntIs, with matches to both protein profiles, were placed in a phylogeny alongside a set of previously identified bacterial IntIs (*23*). IntIs shorter than 200 amino acids or those that did not span the complete IntI-specific domain, needed to distinguish IntIs from other tyrosine recombinases, were removed from phylogenetic analysis. The remaining IntIs were aligned using MAFFT v7.271 (parameters: --localpair --maxiterate 1000) (*59*) and trimmed using trimAl v1.2rev59 (parameters: -automated1). A maximum likelihood tree was generated from the alignment using IQ-TREE v1.6.12 (*56*) with the best-suited protein model as determined by ModelFinder (*60*) and 1000 bootstrap replicates (parameters: -m MFP -bb 1000).

The sequence and structural diversity of *attC*s was assessed using RNAclust v1.3 (*61*) as previously described (*23*). RNAclust uses LocARNA (*62*, *63*) to generate pairwise structural alignments (based on both sequence and folding structure) of input sequences. RNAclust then calculates pairwise distances to create a hierarchical clustering tree from a Weighted Pair Group Method with Arithmetic Mean (WPGMA) analysis. All archaeal *attC*s along with a set of previously identified *attC*s from representative bacterial taxa (*23*) were clustered using RNAclust’s default parameters.

Cassette-encoded proteins identified by IntegronFinder were functionally annotated using InterProScan v5.44-79.0 (*64*), with default parameters against the Pfam (*40*) database, and eggNOG-mapper v2.0.1b (*39*, *65*, *66*), executed in DIAMOND (*67*) mode. To identify cassettes that encode transmembrane and secreted proteins, we searched protein sequences for prokaryotic signal peptides using SignalP 5.0 (*68*) with default parameters. The correlation analysis of cassette functions was performed as described in Penesyan *et al*. (*69*). Briefly, Pearson’s correlations based on co-occurrences among Pfam functions, specific environments, and archaeal phyla were calculated using the Hmisc v4.5-0 R package (*70*). The network was generated from all positive correlations with *P* values of <0.05 using the ForceAtlas2 layout algorithm (*71*) within the Gephi software (*72*). Specific correlations and the description of Pfam functions are listed in data S7.

RBS motifs associated with archaeal cassettes were detected using Prodigal v2.6.3 (*73*) with the implementation of a full RBS motif scan (parameters: -p meta -q -n). To compare RBS motifs against those detected among complete archaeal and bacterial genomes, we downloaded all RefSeq Archaeal genomes (*n* = 443 genomes, 1.2 × 10^6^ genes; downloaded 11 July 2022) and one representative genome from every bacterial order in Reference Sequence (RefSeq) (*n* = 416 genomes, 1.3 × 10^6^ genes; downloaded 11 July 2022). Using Prodigal with the above parameters, RBS motifs were predicted for all archaeal and bacterial genomes.

### Bacterial strains and plasmids for *attC* recombination assays

The bacterial strains and plasmids used in this study are listed in data S8. LB medium (Lennox) was used to grow bacterial strains supplemented with appropriate antimicrobial agents. The final concentrations of antimicrobial agents used were kanamycin (Km; 50 μg/ml), carbenicillin (Cb; 75 μg/ml), and chloramphenicol (Cm; 20 μg/ml). LB medium was supplemented with 0.3 mM 2,6-diaminopimelic (DAP) acid to culture the auxotrophic *E. coli* WM3064 λpir strain (*74*).

### Construction of *attC* donor strains

Nine archaeal *attC*s, selected from diverse archaeal phyla (data S9) along with one bacterial *attC* (*attC_aadA7_*) were used for the recombination assays. Two donor strains were constructed for each *attC*, delivering either the *attC* top or bottom strands via conjugation. Overlapping forward and reverse primers were designed to generate each *attC* sequence flanked by *Xba*I and *Bam*HI overhangs, respectively (e.g., primer pair *attC*-*aadA7*-FW/REV for *attC_aadA7_*). The annealed primer dimers were then ligated into the mobilizable suicide vector pJP5603 (*75*, *76*). The *attC* top strand donor strains were generated by transforming the ligation product into electrocompetent cells of the DAP auxotrophic *E. coli* strain WM3064 λpir. Using the same procedures, all *attC* top strand donor plasmids and strains were constructed using the pairs of long primers listed in data S10.

To deliver *attC* bottom strands, the pJP5603rev (pJPrev) vector was generated to invert *oriT* orientation relative to that of the pJP5603 parental vector. The multiple-cloning site and vector backbone of pJP5603 were PCR-amplified using the primer pairs pJP-MCS-FW/REV and pJP-Backbone-FW/REV, respectively (with *Xho*I and *Mlu*I restriction sites introduced), followed by restriction digest and ligation. The same primer pairs for generating the top strand donor plasmids were used to create the bottom strand donor plasmids and strains by cloning the same *attC* sequences into the *Xba*I/*Bam*HI sites of pJPrev.

### Construction of the recipient strain

We generated a recipient strain using *E. coli* UB5201 (*77*) that carried the *intI1* gene and the *attI1* recombination site residing on the pBAD24 (*78*) and pACYC184 (*79*) backbones, respectively. The *intI1* gene of the R388 plasmid (*80*) was PCR-amplified using the primer pair *intI1_Eco*RI-F/*intI1*_*Hin*dIII-R (data S10). The l-arabinose inducible pBAD::*intI1* plasmid was generated by cloning *intI1* into the pBAD24 expression vector. The pACYC184::*attI1* recipient plasmid was created by assembling the *attI1* sequence (from R388) into the pACYC184 plasmid backbone using the NEBuilder HiFi DNA Assembly Cloning Kit (New England Biolabs, USA). The PCR products required for the assembly were generated using the *attI1*_fw/*attI1*_rev and pACYC184_backbone_F/pACYC184_backbone_R primer pairs. *E. coli* UB5201 strain was cotransformed with pBAD::*intI1* and pACYC184::*attI1* to generate the *E. coli* UB5201 + pBAD::*intI1* + pACYC184::*attI1* recipient strain for *attC* × *attI* suicide conjugation assays. *E. coli* UB5201 + pBAD24 + pACYC184::*attI1* was created as an *intI1*-negative control strain. All plasmid constructs were confirmed by Sanger sequencing and restriction enzyme digests.

### *attC* × *attI* suicide conjugation assays

The frequencies of recombination between the archaeal *attC* sequences and the class 1 integron *attI1* site were quantified using previously established *attC* × *attI* suicide conjugation methods (*26*, *30*, *32*, *81*, *82*) with minor modifications. Briefly, the Cb-resistant UB5201 + pBAD::*intI1* + pACYC184::*attI1* recipient strain was filter-mated with Km-resistant WM3064 λpir *attC* donor strains in DAP-supplemented LB media. The expression of *intI1* was either induced using l-arabinose (2 mg/mL) or suppressed with d-glucose (10 mg/mL). After 6 hours of incubation at 37°C, the recovered conjugation mix was plated on DAP-free LB agar with Km and on LB agar containing Cb. This method was allowed for negative selection of the donor strain, which cannot grow in the absence of DAP, and positive selection of the recombinant recipient clones, which become Km resistant following plasmid cointegration (Fig. 2A). The recombination frequency was determined as the ratio of the colony-forming units (CFUs) for Km-resistant recombinants to the CFU for the total number of Cb-resistant recipients after 2 days of incubation. All assays were performed in three biological replicates, and recombination frequencies were calculated as the mean of the three independent experiments. To confirm the cointegrates, colony PCR was performed on eight randomly chosen colonies per conjugation set for each biological replicate using the following primer pairs: pACYC_F/M13F and pACYC_R/M13R (fig. S3). Sanger sequencing of PCR products was performed for four recombinant colonies per conjugation set.

## Notes

View/request a protocol for this paper from *Bio-protocol*.
