# Supplementary material for: Discovery of integrons in Archaea: Platforms for cross-domain gene transfer
Source: Sci Adv. 2022 Nov 16;8(46):eabq6376. doi: 10.1126/sciadv.abq6376 (PMC9668308; doi:10.1126/sciadv.abq6376)
Supplement: Supplementary file 1 — Table S1 Figs. S1 to S5 [file sciadv.abq6376_sm.pdf]

Science Advances
AAAS

Supplementary Materials for

Discovery of integrons in Archaea: Platforms for cross-domain gene transfer

Timothy M. Ghaly *et al.*

Corresponding author: Timothy M. Ghaly, timothy.ghaly@mq.edu.au

Sci. Adv. **8**, eabq6376 (2022)
DOI: 10.1126/sciadv.abq6376

The PDF file includes:

Table S1
Figs. S1 to S5
Legends for data S1 to S10

Other Supplementary Material for this manuscript includes the following:

Data S1 to S10

Table S1. Average recombination frequencies for the *attC* x *attI* suicide conjugation assays.

	***attC* bottom strand (*intI1* induced*)**	***attC* bottom strand (*intI1* suppressed†)**	***attC* top strand (*intI1* induced)**	***attC* top strand (*intI1* suppressed)**
$attC_{aadA7}$	2.54×10^{-1}	ND††	2.48×10^{-3}	ND
$attC_{\mathrm{Aenigmatarchaeota}}$	5.46×10^{-4}	ND	8.66×10^{-7}	ND
$attC_{\mathrm{Asgardarchaeota\_1}}$	1.79×10^{-2}	ND	6.74×10^{-4}	ND
$attC_{\mathrm{Asgardarchaeota\_2}}$	ND	ND	ND	ND
$attC_{\mathrm{Hadarchaeota}}$	1.13×10^{-1}	ND	1.18×10^{-3}	ND
$attC_{\mathrm{Halobacteriota}}$	4.88×10^{-2}	ND	4.33×10^{-4}	ND
$attC_{\mathrm{Hydrothermarchaeota}}$	5.21×10^{-2}	ND	6.92×10^{-3}	ND
$attC_{\mathrm{Nanoarchaeota}}$	5.84×10^{-2}	ND	1.55×10^{-3}	ND
$attC_{\mathrm{Thermoplasmatota}}$	4.08×10^{-2}	ND	2.28×10^{-3}	ND
$attC_{\mathrm{Thermoproteota}}$	9.54×10^{-2}	ND	1.80×10^{-3}	ND

*induced using L-arabinose; †suppressed using D-glucose; ††ND = Not detected

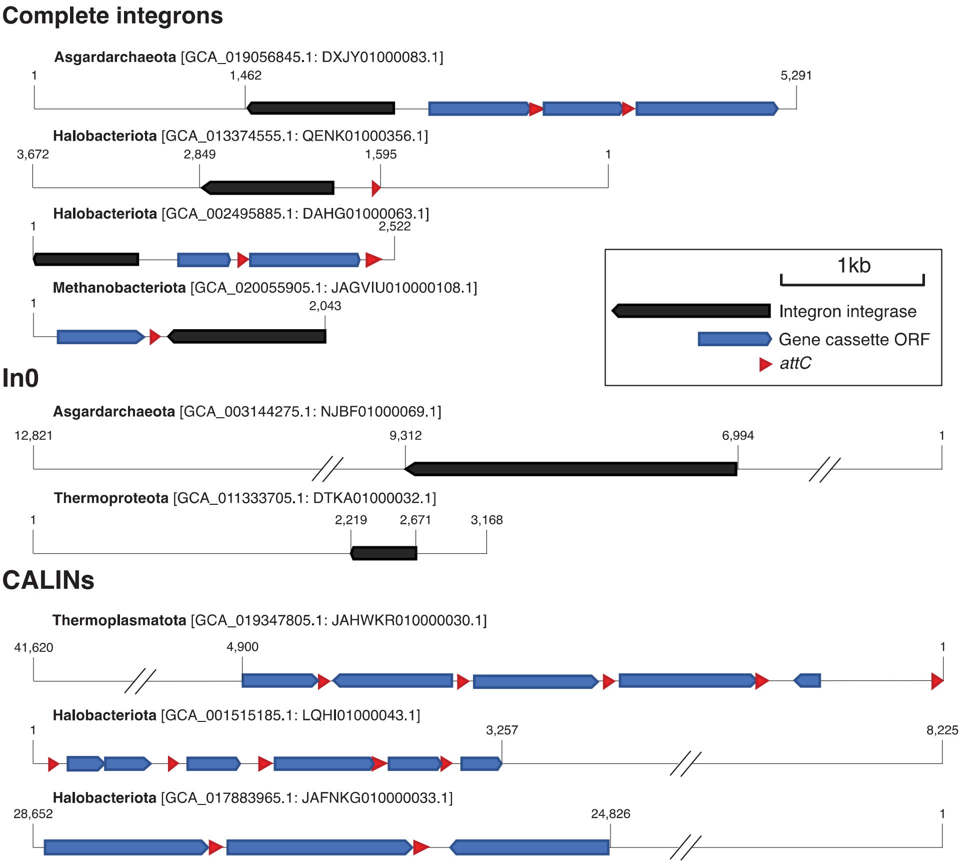

Fig. S1: Example structure of archaeal integrons. Maps of all 'complete integrons', which are those that comprise an integron integrase gene (*intI*) and at least one gene cassette recombination site (*attC*); all 'In0' elements, which are those with *intI* but no detectable *attC* site; and three examples of 'CALINs' (clusters of *attC*s lacking integron integrases).

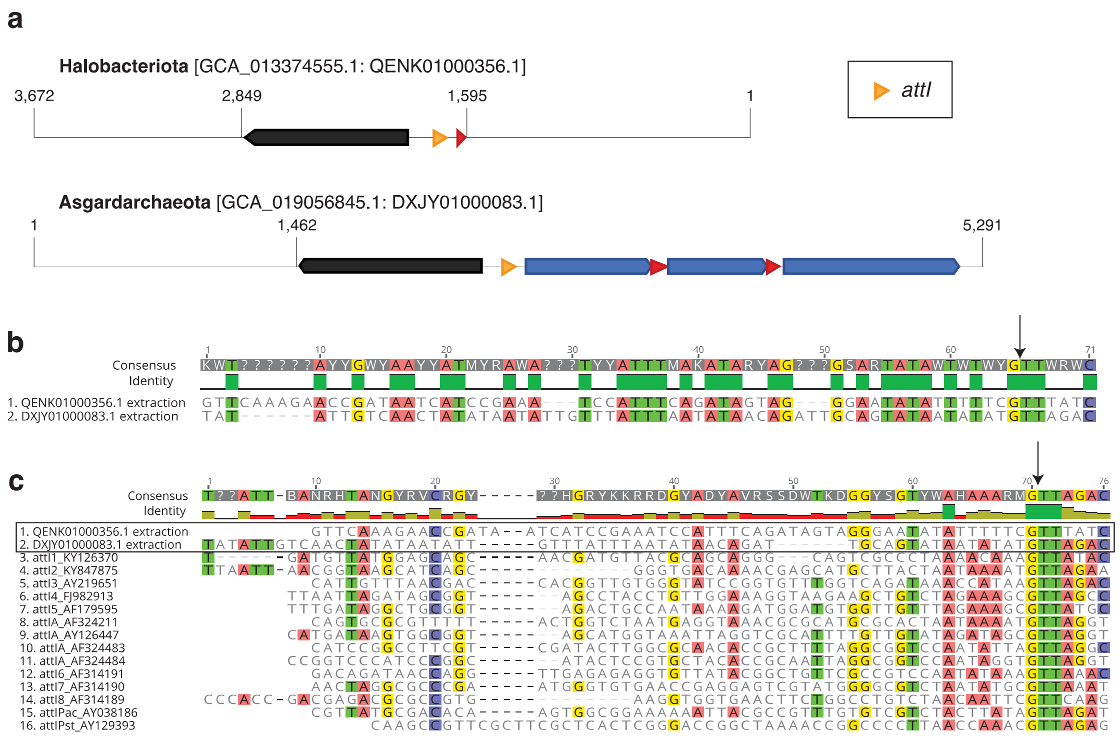

Fig. S2: Putative archaeal integron recombination sites, *attI*s. a, maps showing the location of putative archaeal *attI*s. **b**, sequence alignment of the two putative archaeal *attI*s. **c**, multiple sequence alignment of the two archaeal *attI*s and all annotated bacterial *attI*s from the INTEGRALL database. Nucleotides are coloured if they match with at least 50% of the sequences. Vertical arrows indicate the canonical insertion point of an inserting gene cassette.

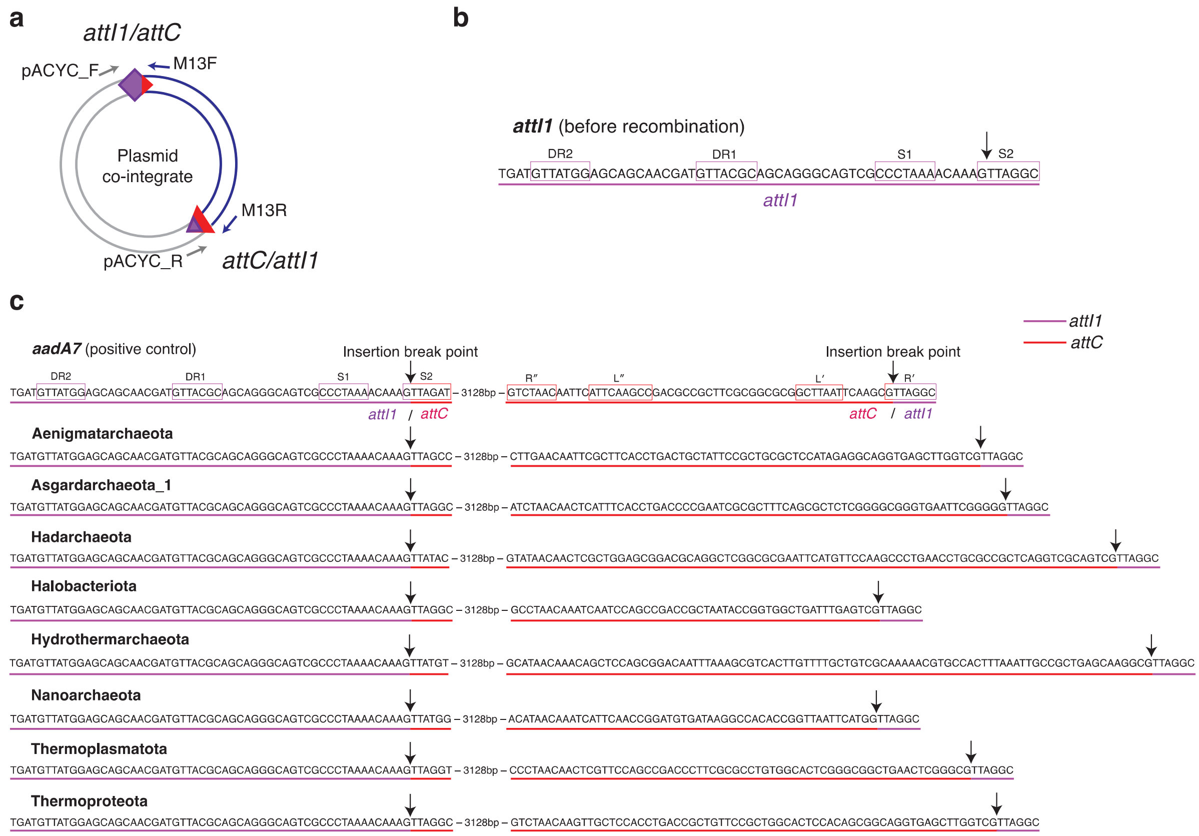

Fig. S3: Sanger sequencing of *attI1* x *attC* recombination junctions. a, schematic of PCR primer pairs (grey and blue arrows) that amplify the recombination junctions following cassette insertion (*attI1* x *attC* recombination). **b,** *attI1* sequence before recombination. Boxes denoted with S1 and S2 indicate the core IntI1 binding sites, and the direct repeats signified by DR1 and DR2, are additional strong and weak IntI1 binding sites, respectively. The black arrow indicates the insertion break point where cleavage takes place during recombination. **c,** Sanger sequence data of the recombinant clones following *attI1* recombination with the paradigmatic bacterial *attC* site ($attC_{aadA7}$), used as positive control, and eight archaeal *attC*s. Black arrows indicate the insertion break points following recombination. For $attC_{aadA7}$, the two sets of paired inverted repeats are boxed (R′ to R″ and L′ to L″).

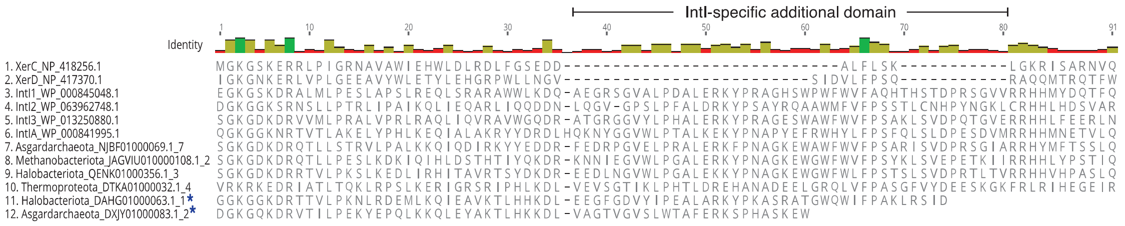

Fig. S4: A multiple protein sequence alignment of the additional domain unique to integron integrases. Sequences (1) and (2) are tyrosine recombinases XerC and XerD that lack the IntI-specific domain. Sequences (3) to (6) are bacterial IntIs, and (7) to (12) are IntIs from Archaea. Blue asterisks indicate IntIs that did not span the full additional domain and were excluded from phylogenetic analysis.

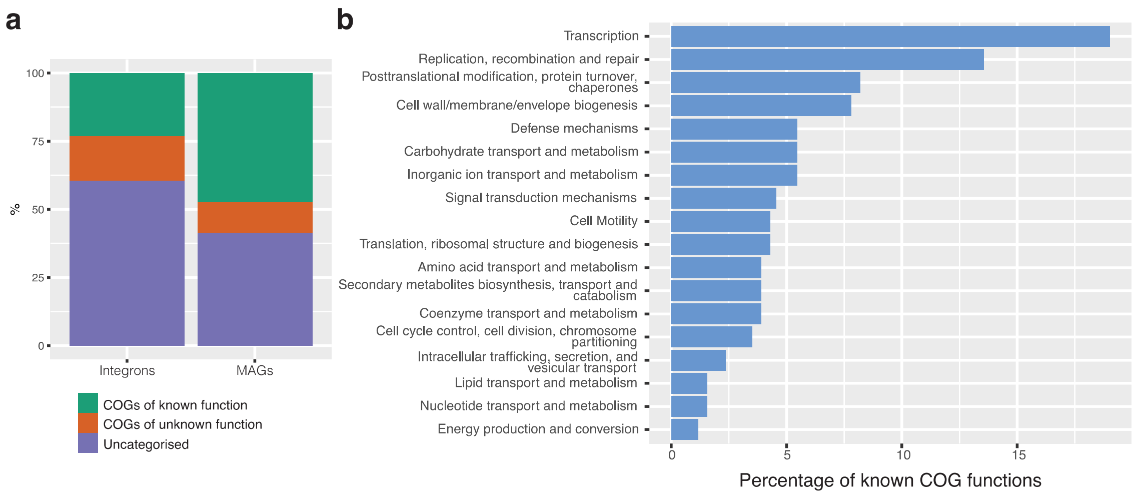

Fig. S5: COG functional analysis of archaeal gene cassettes. a, percentage of proteins assigned a COG category. 'Integrons' represent all cassette-encode proteins in Archaea, while 'MAGs' indicate all proteins from the 75 integron-bearing archaeal genomes. **b**, percentage of COGs with known functions assigned archaeal cassette-encoded proteins.

Title and captions for Data S1 to S10

Data S1. Taxonomy of archaeal genomes with integrons.
Data S2. Identification of prokaryotic marker genes on contigs with integrons.
Data S3. Summary of IntegronFinder output
Data S4. Blast hits of archaeal *attC*s in Bacteria.
Data S5. RBS motifs associated with archaeal integrons and complete archaeal and bacterial genomes
Data S6. Functional predictions of archaeal cassette-encoded proteins.
Data S7. Specific correlations and the description of functions for the Pfam network
Data S8. The bacterial strains and plasmids used in this study
Data S9. Archaeal *attC*s selected for the recombination assays.
Data S10. Primers used for *attC* donor plasmids and strain construction.
